# Individual- and Family-Level Determinants of Risky Sexual Behavior Among Swedish- and Foreign-Born Young Adults 18–30 Years of Age, Residing in Skåne, Sweden

**DOI:** 10.1007/s10508-017-0978-5

**Published:** 2017-05-30

**Authors:** Benedict Oppong Asamoah, Anette Agardh

**Affiliations:** Social Medicine and Global Health, Department of Clinical Sciences, Clinical Research Centre, Lund University, Jan Waldenströms gata 35, House 28, Floor 12, 205 02 Malmö, Sweden

**Keywords:** Sexual behavior, Young people, Family-level determinants, Individual-level determinants, Swedish-born, Foreign-born

## Abstract

In Sweden, various public health interventions have been performed to reduce risky sexual behaviors among young people and promote safer and positive approaches to sexuality, while attempting to bridge the gap between the less privileged or more vulnerable young people and their more privileged peers. This study aimed to compare the individual- and familial-level determinants of risky sexual behavior among foreign-born and Swedish-born young adults 18–30 years of age residing in Skåne, the south of Sweden. This was a cross-sectional study that used a questionnaire to collect data from 2968 randomly selected respondents between 18 and 30 years between January and March 2013. The associations were analyzed using chi-square tests, and simple and multiple logistic regression analyses. Younger age, i.e., individual-level factor, and living with only one parent or another person while growing up, i.e., familial-level factor, increased the risk of engaging in sexual risk taking for both Swedish- and foreign-born youth. Male gender was related to a higher risk of engaging in sexual risk-taking behaviors among foreign-born youth but was not as important as influence on sexual risk taking among Swedish-born youth. Parental education level, on the other hand, was significantly associated with sexual intercourse on the “first night” and early sexual debut solely among Swedish-born youth. Condom use was not associated with any family-level factor among both Swedish-born and foreign-born youth. The design of sexual reproductive health and rights messages and interventions to target risky sexual behavior among Swedish youth should take into consideration immigration status (for example, being Swedish-born or foreign-born), individual- and family-level characteristics, as well as the type of behavioral change or outcome desired.

## Introduction

Sexuality and sexual health among young people are influenced by several factors, some of which are general, although contextual factors also play a crucial role in improving sexual reproductive health and rights (SRHR) of young people (Lazarus et al., 2009). As young people explore different aspects of sexuality, their sexual engagements and outcomes may be influenced by the context in which they grow up, both within and outside of their immediate family environments, locally, nationally, and internationally. These environments could create opportunities for improving sexual and reproductive health of young people by mitigating risks associated with sexual behavior and facilitating a positive approach to sexuality and sexual relationships and the experience of safer sex lives (Elkington et al., 2012; Karim, Magnani, Morgan, & Bond, 2003; World Health Organization, 2011).

Sweden is one of the countries with the most liberal approach to SRHR. Policies and practices allow for and provide young people opportunities to engage in less risky sexual behaviors. However, positive these opportunities may be, contextual factors may influence the extent to which young people belonging to various subgroups in the population benefit from this array of opportunities. Contextual factors can contribute to the creation and propagation of inequality gaps in the way young people experience sexuality and sexual relationships. These factors, when properly addressed with targeted interventions, could help close the gaps in sexual health outcomes for young people with differing backgrounds (Danielsson et al., 2012; Häggström-Nordin, Borneskog, Eriksson, & Tydén, 2011).

The increasing liberal view regarding sex as independent of permanent relationship combined with the fact that more and more people are having children in their later years means that for young people, there is an increasing need to take precautions to avoid unwanted pregnancies and sexually transmitted infections (Danielsson et al., 2012; Herlitz & Forsberg, 2010). Paradoxically, young people tend to have more sexual partners in recent years, with increased prevalence of sexual intercourse on the first “night,” yet condom use has not increased, and the rise in unprotected sexual encounters has resulted in a rise in sexually transmitted infections, chlamydia being the most common (Danielsson et al., 2012). Young people and young adults 15–29 years accounted for 83 percent of all chlamydia cases in Sweden in 2015 (The Public Health Agency of Sweden, 2015). Although the incidence of HIV is generally very low in Sweden, with a skewed incidence rate toward migrants and ethnic groups closely connected to countries with generalized epidemics, the changes in sexual behaviors and the trends shown in other sexually transmitted infections may signify an increasing sexual risk taking among young people in Sweden (Danielsson et al., 2012).

Engaging in first sexual intercourse at an early age has been viewed as a risky sexual behavior (Makenzius & Larsson, 2013), a predictor of risky behaviors (Lara & Abdo, 2016) and a risk factor for poor psychosocial health among young people (Kastbom, Sydsjö, Bladh, Priebe, & Svedin, 2015). Although some studies have argued that early sexual behavior is a determinant of adverse psychosocial health outcomes (Kastbom et al., 2015), Donahue et al. regard the association between early sexual debut and poor psychosocial health as attributable to shared familial influences rather than due to any direct causal association (Donahue, Lichtenstein, Långström, & D’Onofrio, 2013). Possible familial factors that could account for the association between early sexual intercourse and poor psychosocial health could include gene-related personality traits such as sensation seeking and impulsivity (Verweij, Zietsch, Bailey, & Martin, 2009), and environmental factors shared by family members (Dick, Johnson, Viken, & Rose, 2000) such as living in a disadvantaged or unstable household or neighborhood or being raised by parents with low educational status (Donahue et al., 2013; Roche et al., 2005). A thorough understanding of the factors underlying the association between early sexual debut and young people’s adverse psychosocial health could benefit the design of public health interventions. Thus, if the observed association is mainly due to confounding by the influence of familial factors, then aspects such as the family environment and structure need to be considered rather than concentrating public health interventions solely on specific behaviors. Thus, for example, delaying intercourse onset would not effectively reduce adverse outcomes if the association is not causal, but only a reflection of parallel causal factors (Donahue et al., 2013). Donahue et al. found that familial factors accounted for associations between early sexual debut and adolescent child-bearing and criminal offending, whereas non-familial factors accounted for the association between early sexual debut and inconsistent condom use, multiple sexual partners, and increased risk of unwanted pregnancy simply due to an extended number of sexually active years.

Thus, the determinants of early sexual debut and other sexual behaviors among young people could be structured on different levels, such as the individual-level (Shneyderman & Schwartz, 2013), family-level (Wight, Williamson, & Henderson, 2006), institutional-level, and/or other external-level factors (Cavazos-Rehg et al., 2010; McPherson et al., 2013). Such a view is in line with the three interlinked systems described by Kotchick’s ecological model of the determinants of sexual behavior: the self-system (e.g., age, education, and residence), the familial system (e.g., parenting, parental monitoring, and family socioeconomic status), and the extra-familial system (e.g., peers, partners, school, and neighborhood) (Kågesten & Blum, 2015; Kotchick, Shaffer, Miller, & Forehand, 2001).

Factors as diverse as smoking, alcohol use, immigrant background, parental marital status, and parental educational level have been linked to sexual risk taking (Häggström-Nordin et al., 2011; Larsson, Tydén, Hanson, & Häggström-Nordin, 2007). Carlsund and colleagues found that in Sweden, adolescents from single-parent families were about twice as likely to have had sexual debut by age 15 years compared to peers from two-parent families. The odds of early sexual debut in adolescents from families with separated parents having shared physical custody were not significantly higher than in those from two-parent families (Carlsund, Eriksson, Löfstedt, & Sellström, 2013). Parental support has been found to positively influence young people’s well-being, especially adolescents, and lack of support could lead to behavioral problems (Carlsund et al., 2013; Wight et al., 2006).

Being foreign-born, however, presents own opportunities but also challenges for young people. Navigating a “new system” for foreign-born young people could be a challenge and may require a supportive family environment, aside from other forms of support that the extra-familial system provides. Otherwise, the extra-familial system may need to be adapted through targeted interventions that serve the unique needs of these subgroups. For example, girls with foreign backgrounds have been found in previous studies to become sexually active later and to be less likely to use alcohol or to binge drink than girls with a Swedish background (Carlsund et al., 2013; Danielsson et al., 2012). Nevertheless, some studies in Sweden have also found the percentage of abortions among immigrants or children of immigrants to be higher than among native Swedish women (Danielsson et al., 2012).

In Sweden, various public health interventions have been performed to reduce risky sexual behaviors among young people and promote safer and positive approaches to sexuality, while attempting to bridge the gap between the less privileged or more vulnerable young people and their more privileged peers. The healthcare setup and other structures at the national and local levels such as the comprehensive sexuality education in schools and youth friendly clinics could help to provide leverage for high-risk subpopulations. Meanwhile, it is unclear what the impacts of such interventions are for young people with immigrant backgrounds and whether the pattern of individual-level and family-level factors that influence sexual risk taking among young people is similar for both Swedish-born and foreign-born young people. Therefore, this study aimed to compare the individual- and familial-level determinants of risky sexual behavior among foreign-born and Swedish-born young people 18–30 years of age residing in Skåne, the south of Sweden. We hypothesize that although there are shared determinants of risky sexual behavior or experiences among young people in general, some individual- and familial-level determinants (e.g., parent’s education or adult lived with while growing up) may vary based on immigration status in Sweden. In this study, sexual risk-taking behavior was considered to include: non-use of condom at the latest sexual intercourse, sexual intercourse on the “first night” with a previously unknown partner, having 2 or more sexual partners in the last 12 months in Sweden and early sexual debut. These measures were chosen based on the previous studies mentioned above and the potential sexual and reproductive health consequences posed by such experiences.

## Method

### Participants

This cross-sectional study used data from a large population-based survey on sexuality, lifestyle, and health among young people and young adults in Skåne, Sweden, to test the current hypothesis. The survey used a questionnaire with pre-validated questions. The data collection took place between January and March 2013. An invitation to participate was sent out to 7000 randomly selected persons between 18 and 29 years residing in Region Skåne, Sweden, in January 2013. The participants were drawn from the Swedish Population Registry. Potential participants received a letter with information about the study, assuring them of the voluntariness of their participation and anonymity. Detailed description of the study design, study population, and setting can be found elsewhere in a sub-study using data from the same survey (Sundbeck, Emmelin, Mannheimer, Miörner, & Agardh, 2016). The invitation letter included a link by which participants could respond online. Three reminders were sent out, and the last included a printed version of the questionnaire. A total of 2968 persons responded to the questionnaire; among these 2545 (85.7%) reported being Swedish-born and 399 (13.4%) foreign-born. Twenty-four persons (0.8%) who had missing information on place of birth were excluded, resulting in a final sample of 2944 persons. The sample included 29 respondents (1.0%) who reported being 30 years old by the time of filling in the questionnaire.

The questionnaire did not collect information on the country of origin of foreign-born respondents. The immigration pattern in Sweden has changed in the recent years due to the refugee crisis in Europe; however, data from the Swedish Central Population Registry indicate that during the time of data collection for this study (2012/2013), the region of origin for foreign-born residents aged 18–29 in Sweden was mainly Asia (between 40 and 50%), European Union member states (EU 28) excluding the Nordic countries (between 13 and 18%), other European countries excluding EU 28 and the Nordic countries (between 10 and 20%), Africa (between 12 and 17%), the Nordic countries excluding Sweden (4–5%), South America (4–4.5%), and North America (2–2.5%) (SCB, 2016a).

### Measures

#### Independent Variables

##### Individual-Level Factors

*Age* Age was dichotomized as 18–24 years and 25–30 years.

*Sex* Sex was classified as male or female.

##### Family-Level Factors

*Parent’s Birthplace* Parent’s birthplace was derived from the question concerning whether the respondent’s parents were born in Sweden. This question had three response alternatives; “Yes, both,” “No, one of my parents is foreign-born” and “No, both parents are foreign-born.” This was further dichotomized into two categories: “both parents born in Sweden” and “one or both parents born abroad.”

*Adult Lived with While Growing Up* This variable was derived from the question concerning which adults the respondent had lived with for the most part while growing up. The response alternatives were: “my mother and my father,” “my mother,” “my father” and “another person.” This was further dichotomized as “lived with both parents” and “live with one parent or another person.”

*Level of Parent’s Education* This variable was derived from the question concerning the type of education the parent with the highest level of education had. The response alternatives were: “9 years compulsory school,” “2 years of upper secondary school,” “3–4 years of upper secondary school,” “other types of schools” and “university.” This variable was dichotomized as “high” level of education if at least one parent had a university degree, and “low” level of education for all other alternatives.

### Dependent Variables

*Condom Use at Latest Sexual Intercourse* 1 The questionnaire contains several questions about condom use. This variable was derived from the question concerning whether the respondent or their partner had used a condom for protection against sexually transmitted infections during the latest sexual intercourse in Sweden. The response alternatives were: “Yes” for use of condom and “No” for non-use of condom.

*Sexual Intercourse on the “first night”* This variable was derived from the question concerning whether, during the last 12 months in Sweden, the respondent had had sexual intercourse on the “first night” with someone they did not know previously. The response alternatives were: “Yes, once,” “Yes, several times” and “No.” The responses were dichotomized as “Yes” and “No.”

*Number of Sexual Partners* This variable was derived from the question concerning the number of sexual partners the respondent had during the last 12 months in Sweden. The response alternatives were: “0–1,” “2,” “3,” “4 or more.” The responses were dichotomized as “0–1” and “2 or more” based on previous studies.

*Age at First Sexual Intercourse* This variable was derived from the question concerning the age at first sexual intercourse, for those who had sexually debuted. The response alternatives were: “14 years or younger,” “15–17 years,” and “18 years or older.” The responses were dichotomized as: “15 years and older (≥15)” and “below 15 years (≤14).”

### Statistical Analysis

Analyses were performed using Stata version 12.1. The associations between the independent and dependent variables were analyzed using Chi-square tests, and simple and multiple logistic regression analyses. We reported *p* values and odds ratios (ORs) with 95% confidence intervals (CI) (statistical significance level, *α* = 0.05). All variables were adjusted for each other simultaneously in one model. All analyses were done separately for foreign-born and Swedish-born participants.

## Results

Table 1 shows the characteristics of the study sample. Out of the total sample of 2944 participants, 2528 Swedish-born and 397 foreign-born young people aged 18–30 years remained after exclusion of those with missing responses for age and sex. The majority of the Swedish-born respondents (62%) were 18–24 years old compared to the foreign-born youth (48%). Most of the respondents in both groups were females, had lived with both parents while growing up, and had parents with a high educational level. Lack of condom use at latest sexual intercourse was more common among the foreign-born youth (32%) compared to the Swedish-born (22%). Nearly 2 out of 10 (22%) Swedish-born youth and 1 out of 10 foreign-born youth (12%) had had sexual intercourse during the last 12 months on the “first night” with someone who was previously unknown. The proportion of youth who had had 2 or more sexual partners during the last 12 months was 29 and 25% among Swedish- and foreign-born, respectively. Most of the respondents had their sexual debut at 15 years and above. Only 17% of Swedish-born youth and 9% of foreign-born youth had their sexual debut below 15 years of age.

**Table 1:** Distribution of individual- and family-level characteristics, sexual risk-taking experiences for a sample of young Swedish- and foreign-born young adults 18–30 years, residing in Skåne, Sweden (*N* = 2944)

	Swedish-born (*n* = 2545)	Foreign-born (*n* = 399)	*p* value*
Age			<.001
18–24 years	1579 (62.46)	190 (47.86)	
25–30 years	949 (37.54)	207 (52.14)	
Total	2528	397 (100)	
Sex			.480
Male	1049 (41.23)	172 (43.11)	
Female	1495 (58.77)	227 (56.89)	
Total	2544 (100)	399 (100)	
Parents birthplace			<.001
Both parents born in Sweden	2077 (81.84)	36 (9.05)	
One or both parents born abroad	461 (18.16)	362 (90.95)	
Total	2538 (100)	398 (100)	
Adult lived with while growing up			.002
Both parents	1983 (77.98)	281 (70.78)	
One parent or another person	560 (22.02)	116 (29.22)	
Total	2543 (100)	397 (100)	
Parental educational level			.165
Low	995 (39.36)	170 (43.04)	
High	1533 (60.64)	225 (56.96)	
Total	2528 (100)	395 (100)	
Highest educational level			.034
Low	1421 (55.92)	200 (50.25)	
High	1120 (44.08)	198 (49.75)	
Total	2541 (100)	398 (100)	
Non-use of condom at latest sexual intercourse in Sweden			<.001
No	1761 (77.58)	220 (67.48)	
Yes	509 (22.42)	106 (32.52)	
Total	2270	326 (100)	
Sexual intercourse on the “first night”			.003
No	1855 (81.18)	296 (87.83)	
Yes	430 (18.82)	41 (12.17)	
Total	2285 (100)	337 (100)	
2 or more sexual partners			.105
No	1602 (70.14)	248 (74.47)	
Yes	682 (29.86)	85 (25.53)	
Total	2284 (100)	333 (100)	
Early sexual debut (≤14)			<.001
No	1854 (82.51)	293 (90.43)	
Yes	393 (17.49)	31 (9.57)	
Total	2247 (100)	324 (100)	

* p value was produced by Chi-square test and represents statistical significance level, *p* value <.05 = significant

Tables 2 and 3 show the results of the bivariate analysis (Chi-square tests, reported proportions, and *p* values) of the individual- (age and sex) and familial-level (parent’s birthplace, adult lived with while growing up and parental educational level) factors and their association with sexual behavior and experiences among Swedish- and foreign-born youth separately. Younger age and being male were significantly associated with non-use of condom, sex on the “first night,” and 2 or more sexual partners (*p* < .05) irrespective of being Swedish-born or foreign-born. Having one or both parents born abroad was significantly associated with having sex on the “first night” (*p* < .001), 2 or more sexual partners (*p* = .011), and early sexual debut (*p* = .015) among foreign-born youth, whereas the same factor was found to be associated solely with non-use of condom (*p* < .001) among Swedish-born youth. Among both Swedish-born and foreign-born youth, having lived with only one parent or another person while growing up was significantly associated with 2 or more sexual partners (*p* < .001) and early sexual debut (*p* < .001, .003, respectively), whereas the same factor was significantly associated with sex on the “first night” (*p* = .024) only among the foreign-born youth.

**Table 2:** Bivariate analyses of the association between individual- and family-level factors, and sexual risk-taking experiences (non-use of condom and sex on the “first night”) for a sample of Swedish- and foreign-born young adults 18–30 years residing in Skåne, Sweden (*N* = 2944)

	Swedish-born (*n* = 2545)			Foreign-born (*n* = 399)		
	Non-use of condom use at last sexual intercourse in Sweden			Non-use of condom use at last sexual intercourse in Sweden		
	No	Yes	*p* value	No	Yes	*p* value
Age			.001			.029
18–24 years	1017 (75.28)	334 (24.72)		85 (61.15)	54 (38.85)	
25–30 years	773 (81.26)	169 (18.74)		135 (72.58)	51 (27.42)	
Sex			<.001			.003
Male	662 (72.83)	247 (27.17)		82 (58.57)	58 (41.43)	
Female	1099 (80.81)	261 (19.19)		138 (74.19)	48 (25.81)	
Parents birthplace			<.001			.103
Both parents both in Sweden	1501 (79.54)	386 (20.46)		22 (81.48)	5 (18.52)	
One or both parents born abroad	256 (67.72)	122 (32.28)		197 (66.11)	101 (33.89)	
Adult lived with while growing up			.545			.525
Both parents	1360 (77.27)	400 (22.73)		153 (66.23)	78 (33.77)	
One parent or another person	399 (78.54)	109 (21.46)		65 (69.89)	28 (30.11)	
Level of parent’s education			.089			.205
Low	720 (79.38)	187 (20.62)		91 (64.08)	51 (35.92)	
High	1029 (76.34)	319 (23.66)		128 (70.72)	53 (29.28)	

	Sexual intercourse on the “first night”			Sexual intercourse on the “first night”		
	No	Yes	*p* value	No	Yes	*p* value
Age			<.001			<.001
18–24 years	1072 (78.65)	291 (21.35)		116 (79.45)	30 (20.55)	
25–30 years	769 (84.88)	137 (15.12)		178 (94.18)	11 (5.82)	
Sex			<.001			.012
Male	698 (76.37)	216 (23.63)		119 (82.64)	25 (17.36)	
Female	1157 (81.22)	213 (15.55)		177 (91.71)	16 (8.29)	
Parents birthplace			.331			<.001
Sweden (both parents)	1542 (81.50)	350 (18.50)		16 (61.54)	10 (38.46)	
One or both born abroad	308 (79.38)	80 (20.62)		279 (90.00)	31 (10.00)	
Adult lived with while growing up			.069			.024
Both parents	1458 (81.00)	320 (18.00)		215 (90.34)	23 (9.66)	
One parent or another person	396 (78.42)	109 (21.58)		79 (81.44)	18 (18.56)	
Level of parent’s education			<.001			.990
Low	774 (84.87)	138 (15.13)		132 (88.00)	18 (12.00)	
High	1068 (78.78)	289 (21.30)		162 (88.04)	22 (11.96)

**Table 3:** Bivariate analyses of the association between the individual- and family-level factors, and sexual risk-taking experiences (2 or more sexual partners and early sexual debut) for a sample of Swedish- and foreign-born young adults 18–30 years residing in Skåne, Sweden (*N* = 2944)

	Swedish-born (*n* = 2545)			Foreign-born (399)		
	2 or more sexual partners			2 or more sexual partners		
	No	Yes	*p* value	No	Yes	*p* value
Age			<.001			<.001
18–24 years	888 (65.34)	471 (34.66)		92 (64.79)	50 (35.21)	
25–30 years	699 (76.98)	209 (23.02)		156 (82.11)	34 (17.89)	
Sex			.534			.006
Male	634 (69.44)	279 (30.56)		95 (66.90)	47 (33.10)	
Female	968 (70.66)	402 (29.34)		153 (80.10)	38 (19.90)	
Parents birthplace			.173			.011
Both parents born in Sweden	1337 (70.67)	555 (29.33)		14 (53.85)	12 (46.15)	
One or both parents born abroad	260 (67.18)	127 (32.82)		234 (76.47)	72 (23.53)	
Adult lived with while growing up			<.001			<.001
Both parents	1276 (71.97)	497 (28.03)		188 (80.00)	47 (20.00)	
One parent or another person	325 (63.85)	184 (36.15)		58 (60.42)	38 (39.58)	
Level of parent’s education			.061			.396
Low	662 (72.35)	253 (27.65)		111 (76.55)	34 (23.45)	
High	930 (68.69)	424 (31.31)		134 (72.43)	51 (27.57)	

	Early sexual debut			Early sexual debut		
	No (≥15 years)	Yes (≤14 years)	*p* value	No (≥15 years)	Yes (≤14 years)	*p* value
Age			.187			.293
18–24 years	1086 (81.59)	245 (18.41)		122 (88.41)	16 (11.59)	
25–30 years	753 (83.76)	146 (16.24)		170 (91.89)	15 (8.11)	
Sex			.285			.541
Male	747 (83.56)	147 (16.44)		125 (89.29)	15 (10.71)	
Female	1106 (81.80)	246 (18.20)		168 (91.30)	16 (8.70)	
Parents birthplace			.886			.015
Both parents born in Sweden	1544 (82.57)	326 (17.43)		20 (76.92)	6 (23.08)	
One or both parents born abroad	306 (82.52)	66 (17.74)		272 (91.58)	25 (8.42)	
Adult lived with while growing up			<.001			.003
Both parents	1482 (84.83)	265 (15.17)		214 (93.45)	15 (6.55)	
One parent or another person	371 (74.35)	128 (25.65)		77 (82.80)	16 (17.20)	
Level of parent’s education			<.001			.890
Low	708 (79.11)	187 (20.89)		127 (90.71)	13 (9.29)	
High	1134 (84.88)	389 (17.44)		165 (91.16)	16 (8.84)	

Tables 4 and 5 show the results from the simple and multiple logistic regression analysis modeling the association between the individual- and familial-level background factors and risky sexual behavior (non-use of condom and having sex on the “first night” [Table 4], 2 or more sexual partners and early sexual debut [Table 5]) among Swedish- and foreign-born youth. The results revealed similar associations as those in the chi-square tests shown in Tables 2 and 3.

**Table 4:** Simple and multiple logistic regression analyses of the association (odds ratio (OR) and 95% confidence interval (CI)) between individual- and family-level factors, and sexual risk-taking experiences (non-use of condom and sex on the “first night”) for a sample of Swedish- and foreign-born young adults 18–30 years residing in Skåne, Sweden (*N* = 2944)

	Swedish-born (2545)		Foreign-born (399)	
	Crude OR (95% CI)	Adjusted OR* (95% CI)	Crude OR (95% CI)	Adjusted OR* (95% CI)
*Non-use of condom at latest sexual intercourse*				
Age				
18–24 years	1.4 (1.2–1.8)	1.4 (1.1–1.7)	1.7 (1.1–2.7)	1.9 (1.1–3.1)
25–30 years	Ref	Ref	Ref	Ref
Sex				
Male	1.6 (1.3–1.9)	1.6 (1.3–1.9)	2.0 (1.3–3.3)	1.9 (1.2–3.2)
Female	Ref	Ref	Ref	Ref
Parents birthplace				
Both parents born in Sweden	Ref	Ref	Ref	Ref
One or both parents born abroad	1.9 (1.5–2.4)	1.8 (1.4–2.3)	2.3 (0.8–6.1)	2.5 (0.9–7.0)
Adult lived with while growing up				
Both parents	Ref	Ref	Ref	Ref
One parent or another person	0.9 (0.7–1.2)	0.9 (0.7–1.2)	0.8 (0.5–1.4)	0.7 (0.4–1.2)
Level of parent’s education				
Low	1.2 (1.0–1.5)	0.9 (0.7–1.1)	0.7 (0.5–1.2)	1.4 (0.8–2.3)
High	Ref	Ref	Ref	Ref
*Sex on the “first night” with a previously unknown person*				
Age				
18–24 years	1.5 (1.2–1.9)	1.5 (1.2–1.9)	4.2 (2.0–8.7)	3.3 (1.5–7.1)
25–30 years	Ref	Ref	Ref	Ref
Sex				
Male	1.7 (1.4–2.1)	1.7 (1.4–2.1)	2.3 (1.2–4.5)	2.6 (1.3–5.4)
Female	Ref	Ref	Ref	Ref
Parents birthplace				
Both parents born in Sweden	Ref	Ref	Ref	Ref
One or both parents born abroad	1.1 (0.9–1.5)	1.0 (0.8–1.4)	0.2 (0.1–0.4)	0.1 (0.1–0.4)
Adult lived with while growing up				
Both parents	Ref	Ref	Ref	Ref
One parent or another person	1.3 (1.0–1.6)	1.3 (1.0–1.7)	2.1 (1.1–4.2)	1.9 (0.9–4.0)
Level of parent’s education				
Low	0.7 (0.5–0.8)	0.6 (0.5–0.8)	1.0 (0.5–2.0)	1.3 (0.6–2.7)
High	Ref	Ref	Ref	Ref

* All variables were simultaneously adjusted for one another

**Table 5:** Simple and multiple logistic regression analyses of the association (odds ratio (OR) and 95% confidence interval (CI)) between individual- and family-level factors, and sexual risk-taking experiences (2 or more sexual partners and early sexual debut) for a sample of Swedish- and foreign-born young adults 18–30 years residing in Skåne, Sweden (*N* = 2944)

	Swedish-born		Foreign-born	
	Crude OR (95% CI)	Adjusted OR* (95% CI)	Crude OR (95% CI)	Adjusted OR* (95% CI)
*2 or more sexual partners in the last 12* *months in Sweden*				
Age				
18–24 years	1.8 (1.5–2.1)	1.8 (1.5–2.1)	2.5 (1.5–4.1)	2.1 (1.2–3.6)
25–30 years	Ref	Ref	Ref	Ref
Sex				
Male	1.1 (0.9–1.3)	1.1 (0.9–1.3)	2.0 (1.2–3.3)	1.9 (1.1–3.2)
Female	Ref	Ref	Ref	Ref
Parents birthplace				
Both parents born in Sweden	Ref	Ref	Ref	Ref
One or both parents born abroad	1.2 (0.9–1.5)	1.1 (0.9–1.4)	0.4 (0.2–0.8)	0.3 (0.1–0.8)
Adult lived with while growing up				
Both parents	Ref	Ref	Ref	Ref
One parent or another person	1.5 (1.2–1.8)	1.5 (1.2–1.9)	2.6 (1.6–4.4)	2.7 (1.6–4.8)
Level of parent’s education				
Low	0.8 (0.7–1.0)	0.8 (0.7–1.0)	0.8 (0.5–1.3)	0.9 (0.5–1.6)
High	Ref	Ref	Ref	Ref
*Early sexual debut (≤14* *years)*				
Age				
18–24 years	1.2 (0.9–1.5)	1.2 (1.0–1.5)	1.5 (0.7–3.1)	1.0 (0.4–2.1)
25–30 years	Ref	Ref	Ref	Ref
Sex				
Male	0–9 (0.7–1.1)	0.9 (0.7–1.1)	1.3 (0.6–2.6)	1.6 (0.7–3.5)
Female	Ref	Ref	Ref	Ref
Parents birthplace				
Both parents born in Sweden	Ref	Ref	Ref	Ref
One or both parents born abroad	1.0 (0.8–1.4)	0.9 (0.7–1.2)	0.3 (0.1–0.8)	0.2 (0.1–0.6)
Adult lived with while growing up				
Both parents	Ref	Ref	Ref	Ref
One parent or another person	1.9 (1.5–2.5)	1.8 (1.4–2.3)	3.0 (1.4–6.3)	3.3 (1.5–7.5)
Level of parent’s education				
Low	1.5 (1.2–1.8)	1.4 (1.1–1.8)	1.1 (0.5–2.3)	1.2 (0.5–2.7)
High	Ref	Ref	Ref	Ref

* All variables were simultaneously adjusted for one another

The likelihood of individual- and familial-level factors determining risky sexual behaviors and experiences was generally high for the foreign-born respondents compared to their Swedish-born peers. Younger respondents aged 18–24 were significantly more likely among both Swedish-born and foreign-born not to use a condom during their latest sexual intercourse during the last 12 months (OR_adjusted_ 1.4 and 1.9, respectively), to have sexual intercourse with a previously unknown person on the “first night” (OR_adjusted_ 1.4 and 3.3, respectively) and to have 2 or more sexual partners during the last 12 months (OR_adjusted_ 1.8 and 2.1, respectively), although the magnitude of the risk varied within each group (Tables 4, 5). Being a young male was significantly associated with a high risk of engaging in sex without a condom (OR_adjusted_ 1.6 and 1.9, respectively) and having sexual intercourse with a previously unknown person on the “first night” (OR_adjusted_ 1.7 and 2.6, respectively) in both Swedish- and foreign-born youth, but significantly associated with 2 or more partners only among the foreign-born youth (OR_adjusted_ 1.9). Compared with peers who lived with both parents while growing up, living with only one adult (parent or another person) while growing up had no association with condom use at latest sexual intercourse among both Swedish-born and foreign-born, but significantly increased the risk of having sex on the “first night” for Swedish-born youth (OR_adjusted_ 1.3), and for having 2 or more sexual partners in the last 12 months (OR_adjusted_ 1.5 and 2.7, respectively), and for early sexual debut (OR_adjusted_ 1.8 and 3.3, respectively) for both Swedish- and foreign-born.

Parental education level was not found to play any significant role concerning any sexual behavior among foreign-born youth, nor did it play a role in condom use and number of sexual partners among Swedish-born youth, but had dynamic associations with sexual intercourse on the “first night” and early sexual debut among Swedish-born youth. Whereas Swedish-born youth whose parents have a low-level education had about 40% reduced risk of having sex on the “first night” with a previously unknown person (OR_adjusted_ 0.6), they had a 40% increased risk of early sexual debut (OR_adjusted_ 1.4) compared to their peers whose parents have high educational level.

## Discussion

The hypothesis for this study was generally supported by the results obtained. Thus, the determinants of risky sexual behavior or experiences among foreign-born and Swedish-born respondents were not generally different across all the predictors that were tested, but these two groups differed rather on some specific determinants of risky sexual behavior. This study found that in the fully adjusted model the individual-level factor, i.e., younger age, and the family-level factor, i.e., living with only one parent or another person while growing up, posed a higher risk of engaging in sexual risk taking for both Swedish- and foreign-born youth. However, the magnitude of the effect represented by these factors with regard to sexual risk taking was stronger among foreign-born youth. For other factors, both at the individual and family levels, the pattern was entirely different. For example, male gender was related to a higher risk of engaging in sexual risk-taking behaviors among foreign-born youth but was not as important as influence on sexual risk taking among Swedish-born youth. Parental education level, on the other hand, was significantly associated with sexual intercourse on the “first night” and early sexual debut solely among Swedish-born youth. Condom use was not associated with any family-level factor among both Swedish-born and foreign-born youth.

The results from this study are largely in accordance with recent reviews from a variety of different settings (McPherson et al., 2013; Mmari & Sabherwal, 2013). The review by McPherson et al. indicated that thirteen studies showed evidence that living with at least one biological parent was an important protective factor for sexual risk taking and adverse sexual health outcomes. In the current study, living with one parent or another person while growing up was found to be associated with higher number of partners and early sexual debut in both groups and sexual intercourse on the “first night” among Swedish-born youth. This is also supported by previous studies in Sweden that found that family structure and culture matters predicted age of sexual debut (Kastbom, Sydsjö, Bladh, Priebe, & Svedin, 2016). Kastbom et al. found that Swedish adolescents living with both biological parents or alternating between them were more likely to postpone their sexual debut. Other studies in both western and non-western countries have previously found that living in a two-parent family was a protective factor for young people (Haglund & Fehring, 2010; Potdar & Mmari, 2011), whereas impaired families or unstable family environment was associated with risky sexual behavior and early age of sexual debut (Goldberg, 2013; Sheppard, Garcia, & Sear, 2014; Tsitsika et al., 2010).

As shown in this study, condom use is influenced by an array of factors that transcend the family system, irrespective of immigration status. Being a male or younger plays a role in condom use in both Swedish- and foreign-born youth, although more strongly among foreign-born youth, as shown in this study. This is supported by previous studies in both Sweden and abroad (Ekstrand, Larsson, Von Essen, & Tydén, 2005). However, being a male does not seem to play any significant role in the number of sexual partners that Swedish-born youth have but plays a rather significant role among foreign-born youth. This could be due to strong gender-related norms (male dominance) and culture regarding sexual relations among foreign-born respondents that may not be present among Swedish-born respondents. As mentioned above, the adjusted results from this study showed that individual-level factors influenced condom use. Although this study did not examine all the different dimensions of individual-level factors, previous studies suggest that the decision to use a condom is primarily influenced by the person’s individual circumstances, including risk perceptions of acquiring a sexually transmitted infection, trust in partners, relationship status, and other perceived benefits of using a condom, weighed against the perception of reduced pleasure and sensitivity with condom use (Ekstrand et al., 2005; Romero-Estudillo, González-Jiménez, Mesa-Franco, & García-García, 2014). However, Wight et al. also found that low parental monitoring among Scottish youth was associated with less consistent condom and contraception use by females but not males (Wight et al., 2006).

One issue that could be of concern for SRHR interventions is that parental education seemingly plays a role only among Swedish-born and not among foreign-born youth. The previous study by Wight et al. among Scottish young people also found that Scottish parents with higher education were more likely to talk with their children about sex, whereas immigrant parents such as Indians and Pakistanis with similar educational background were less likely to do so. One explanation for this could be that regardless of educational level, immigrant parents might not be able to discuss sex with their “offspring” due to cultural norms prohibiting this type of discourse (Ayehu, Kassaw, & Hailu, 2016; Roudsari, Javadnoori, Hasanpour, Hazavehei, & Taghipour, 2013; Sridawruang, Pfeil, & Crozier, 2010). The finding in this current study that among Swedish-born youth higher parental education is protective against early sexual debut but inversely predicts sexual intercourse on the “first night” with a previously unknown person is counter intuitive. A possible reason behind this could be that having higher parental education may be associated with greater sexual knowledge, self-efficacy, and/or assertiveness. Thus, individuals with higher parental education may abstain from sex until they feel ready to have it and then be more confident/willing to have it when they are ready. Whatever the reason may be, such findings support the previous evidence that the most promising sexual and reproductive health and rights interventions for preventing multiple risk behaviors are those that addressed multiple aspects (individual and peer, family, school and community) of risk-promoting and risk-protective factors for risk behavior (Jackson, Geddes, Haw, & Frank, 2012).

### Methodological Considerations

This was a cross-sectional study and thus has limitations with regard to inferring causation. However, because of the temporality between the independent and dependent measures used in this study, the possible explanation of a reverse causation can be excluded.

Recall bias is a possible limitation, due to the retrospective nature of the questions. We tried to reduce recall bias for the three dependent variables: non-use of condom, number of sexual partners, and sex on the “first night” by limiting the condom use question to the last sexual experience in Sweden, and the remaining two questions to sexual experiences during the last 12 months. However, the possibility of some recall bias still remains. The question concerning age at first sexual debut had three response alternatives in the original questionnaire. However, a possibility of social desirability bias exists, especially for foreign-born youth originating from settings with cultural norms that strongly prohibit early sexual intercourse, who might be reluctant to admit early debut. Although this is possible, due to the assurance of anonymity of responses, we regard this as a very minimal source of bias.

Another limitation worth considering is that this study does not compare foreign-born with Swedish-born but rather examined subgroups within the categories Swedish-born and foreign-born. However, on the other hand, this could be considered as advantageous for this study, because making such comparisons would also limit our analysis regarding exploring the individual- and family-level determinants of sexual behavior in groups. Thus, caution should be taken in interpreting the current results.

The possibility of selection bias could arise from the remuneration used to increase participation, although the cinema ticket given to compensate for participant’s time spent in responding to the questionnaire is valued at such low cost that it is not likely to have caused a differential response from any group of participants.

The proportion of foreign-born young people 18–30 years who participated in the survey was limited (about 13%). This resulted in slightly wider confidence intervals for the analysis conducted within the foreign-born subgroup. Data from the Swedish Central Population Registry indicate that in 2012/2013, the proportion of foreign-born young persons residing in Skåne aged 15–24 and 25–34 years was around 15 and 30%, respectively (SCB, 2016b). Looking at the above statistics, 13% could be considered to be a fairly good participation rate for the age group 18–29 years. The sample selection process excluded undocumented foreign-born young adults, which could have resulted in underestimation of the individual- and family-level determinants of sexual risk-taking behaviors among the foreign-born young adults.

Lastly, the information about the adult with whom the participant lived while growing up was limited, and it is not known, for example, whether parents were biological or same-sex parents.

### Conclusions

Among both Swedish-born and foreign-born youth, both individual and context-specific family-level factors acted as determinants of risky sexual behaviors. Individual-level factors such as younger age and male sex were found to generally predict sexual risk-taking behaviors among both Swedish-born and foreign-born youth.

Whereas some sexual risk-taking behaviors such as having sexual intercourse at the first night with a person previously unknown and higher number of sexual partners had both individual and family-level determinants, condom use was solely found to be associated with individual-level predictors, whereas age at sexual debut was solely found to be associated with family-level predictors. Thus, the design of sexual reproductive health and rights (SRHR) messages and interventions to target risky sexual behavior among Swedish youth should take into consideration immigration status (for example, being Swedish-born or foreign-born), individual- and family-level characteristics, as well as the type of behavioral change or outcome desired.

## Footnotes

1 Participants were asked about the use of a condom to prevent unwanted pregnancy, but these analyses focus on condom use to prevent sexually transmitted infections (STIs).
